# MEK1-ERK1/2 signaling regulates the cardiomyocyte non-sarcomeric actin cytoskeletal network

**DOI:** 10.1152/ajpheart.00612.2023

**Published:** 2023-11-24

**Authors:** Kelly M. Grimes, Marjorie Maillet, Casey O. Swoboda, Stephanie L. K. Bowers, Doug P. Millay, Jeffery D. Molkentin

**Affiliations:** Department of Pediatrics, Cincinnati Children’s Hospital Medical Center, University of Cincinnati, Cincinnati, Ohio, United States

**Keywords:** cardiomyocyte, cytoplasmic actin, cytoskeleton, hypertrophy, MAPK signaling

## Abstract

During select pathological conditions, the heart can hypertrophy and remodel in either a dilated or concentric ventricular geometry, which is associated with lengthening or widening of cardiomyocytes, respectively. The mitogen-activated protein kinase kinase 1 (MEK1) and extracellular signal-related kinase 1 and 2 (ERK1/2) pathway has been implicated in these differential types of growth such that cardiac overexpression of activated MEK1 causes profound concentric hypertrophy and cardiomyocyte thickening, while genetic ablation of the genes encoding ERK1/2 in the mouse heart causes dilation and cardiomyocyte lengthening. However, the mechanisms by which this kinase signaling pathway controls cardiomyocyte directional growth as well as its downstream effectors are poorly understood. To investigate this, we conducted an unbiased phosphoproteomic screen in cultured neonatal rat ventricular myocytes treated with an activated MEK1 adenovirus, the MEK1 inhibitor U0126, or an eGFP adenovirus control. Bioinformatic analysis identified cytoskeletal-related proteins as the largest subset of differentially phosphorylated proteins. Phos-tag and traditional Western blotting were performed to confirm that many cytoskeletal proteins displayed changes in phosphorylation with manipulations in MEK1-ERK1/2 signaling. From this, we hypothesized that the actin cytoskeleton would be changed in vivo in the mouse heart. Indeed, we found that activated MEK1 transgenic mice and gene-deleted mice lacking ERK1/2 protein had enhanced non-sarcomeric actin expression in cardiomyocytes compared with wild-type control hearts. Consistent with these results, cytoplasmic β- and γ-actin were increased at the subcortical intracellular regions of adult cardiomyocytes. Together, these data suggest that MEK1-ERK1/2 signaling influences the non-sarcomeric cytoskeletal actin network, which may be important for facilitating the growth of cardiomyocytes in length and/or width.

**NEW & NOTEWORTHY** Here, we performed an unbiased analysis of the total phosphoproteome downstream of MEK1-ERK1/2 kinase signaling in cardiomyocytes. Pathway analysis suggested that proteins of the non-sarcomeric cytoskeleton were the most differentially affected. We showed that cytoplasmic β-actin and γ-actin isoforms, regulated by MEK1-ERK1/2, are localized to the subcortical space at both lateral membranes and intercalated discs of adult cardiomyocytes suggesting how MEK1-ERK1/2 signaling might underlie directional growth of adult cardiomyocytes.

## INTRODUCTION

The mitogen-activated protein kinase (MAPK) pathway is a phosphorylation amplification cascade that transduces a wide array of growth and stress-responsive signals in nearly all cell types (1–3). Growth factors and their receptors located within the plasma membrane initiate cytoplasmic Ras/Raf signaling leading directly to the phosphorylation of MEK1/2 (mitogen-activated protein kinase kinase 1 and 2), which are dedicated dual specificity kinases for ERK1/2 (extracellular signal-related kinase 1 and 2), on which they phosphorylate TEY motifs to cause activation (4). ERK1/2 are serine/threonine kinases that have a diverse array of intracellular targets that directly mediate stress and growth responses, although the downstream effectors of ERK1/2 [beyond the classical nuclear targets of ELK-1, c-FOS, p53, GATA4, and ETS1/2 (5)] in cardiomyocytes are widely unknown.

We have previously shown that MEK1-ERK1/2 signaling regulates the ability of cardiomyocytes to grow in length or width, shifting the balance toward dilated or concentric cardiac hypertrophy depending on the activity of this pathway (6). For example, mice lacking the genes for ERK1/2 (*Mapk1/3*) in cardiomyocytes showed profound ventricular dilation with cardiomyocytes that grew in length (6). Conversely, hearts of transgenic mice expressing an activated form of MEK1 in cardiomyocytes showed constitutive phosphorylation of ERK1/2, ventricular concentric remodeling, and only thickening of cardiomyocytes (7). These same two effects of MEK1-ERK1/2 signaling were shown to underlie concentric versus dilated ventricular remodeling in the genetic cardiomyopathies, as reported across a spectrum of mice that modeled hypertrophic and dilated cardiomyopathy (8). We hypothesized that conducting a phosphoproteomic screen under conditions of altered MEK1-ERK1/2 signaling would provide insight into the effectors that might selectively alter the growth properties of cardiomyocytes.

As much as 30% of ERK1/2 proteins in a cell are tethered to components of the cytoskeleton, as well as localized to focal adhesion complexes (4). While MEK1-ERK1/2 signaling underlies cardiomyocyte growth dynamics, and this can correlate with changes in cytoskeletal proteins (9, 10), very little is known of cardiomyocyte-specific phosphorylation targets of ERK1/2, let alone cytoskeletal protein targets. The cytoskeleton comprises 3 major interdependent components: microtubules, intermediate filaments, and actin microfilaments. There are 6 isoforms of actin expressed in the heart: sarcomeric cardiac α-actin (*Actc1*) and skeletal α-actin (*Acta1*), the cytoplasmic β- and γ-actins (*Actb* and *Actg1*, respectively), and the smooth muscle α- and γ-actins (*Acta2* and *Actg2*, respectively) (11). Beyond the sarcomeric α-actin network within the contractile thin filaments, cardiomyocytes also express the cytoplasmic actins (12). We know from studies in skeletal myocytes that this network localizes to the subcortical space (13); however, reports on the localization of this network in cardiomyocytes are conflicted (14, 15). In addition to the actin of the sarcomeres and cytoplasmic network, cardiomyocytes also contain intermediate filaments and microtubules (16, 17). Although much research has been conducted on the microtubules and the desmin intermediate filament networks in cardiomyocytes, the function and dynamics of the non-sarcomeric cytoplasmic actin network have been difficult to study given the overwhelming content of contractile sarcomeric α-actin that obscures detection of cytoplasmic actin isoforms (18). However, mutations in cytoplasmic actin-associated proteins can cause cardiomyopathies (15, 19, 20), suggesting that the actin that resides outside the sarcomeric network is an important area of cardiomyocyte biology that necessitates further study. Upon analyzing the data of the phosphoproteomic screen conducted here, we hypothesized that changes in MEK1-ERK1/2 signaling would alter the cardiomyocyte cytoskeleton in vivo as well as in vitro.

## MATERIALS AND METHODS

### Cell Culture

Neonatal rat ventricular myocytes (NRVMs) were isolated from hearts of 1- to 2-day-old male and female rat pups born to timed-mated Sprague-Dawley dams (Envigo, Cat. No. 002). The atria were removed, and the ventricles were minced in HBSS before overnight enzymatic digestion using trypsin (Worthington, TRLS). After trypsin inhibition (Worthington, SIC) for 15 min at 37°C followed by collagenase treatment (Worthington, CLSPA) for 1 h at 37°C, cells were collected by centrifugation at 1,000 *g* for 5 min and resuspended in M199 medium (Thermo Fisher Scientific, Cat. No. SH30253FS). The cells were then differentially plated for 2 h on culture dishes to reduce fibroblast content. Cell culture dishes were coated with 0.1% gelatin (Bio-Rad, Cat. No. 170-6537) before plating the cells in M199 medium supplemented with 15% bovine growth serum (BGS; Thermo Fisher, Cat. No. SH3054103) and 1× penicillin-streptomycin (Cellgro, Cat. No. 30-0002-CI). NRVMs were incubated at 37°C in 5% CO_2_ overnight and then switched into M199 medium with 1% BGS, followed by infection with β-galactosidase, enhanced green fluorescent protein (eGFP), or activated MEK1 recombinant adenovirus for 2.5 h, and then switched into virus-free M199 medium with 1% BGS and allowed to incubate for 36 h. Cells were then switched to serum-free medium for 24 h. At the end of this period, one cohort of NRVMs not infected with adenoviruses was treated with 10 µM U0126 (Promega, Cat. No. V1121) for 1 h in serum-free M199 before fixation or homogenization. The β-galactosidase, eGFP, and activated MEK1 adenoviruses were produced and applied to NRVMs, as previously described (7).

### Phosphoproteomics

NRVMs were prepared for PTMScan mass spectrometry analysis, as previously described (21). NRVMs were washed twice with ice-cold 1× phosphate-buffered saline (PBS), and scraped into urea lysis buffer, consisting of 9 M sequanal grade urea, 20 mM HEPES (pH 8.0), 1 mM sodium vanadate, 1 mM β-glycerophosphate, and 2.5 mM sodium pyrophosphate. NRVM extracts were sonicated, centrifuged, reduced with DTT, and alkylated with iodoacetamide. Equal amounts of total protein for each sample were digested with trypsin, purified over C18 columns (Waters), and proline-based phosphopeptide enrichment was performed using the following motif antibodies: MAPK substrate motif antibody (Cell Signaling, Cat. No. 2325), tP and tPE motif antibody (Cell Signaling, Cat. No. 3004), and StP motif antibody (Cell Signaling, Cat. No. 5566). LC-MS/MS analysis was performed using an LTQ-Orbitrap-Velos, ESI-CID (Thermo Fisher). Peptides were loaded directly onto a 10 cm × 75 μm PicoFrit capillary column packed with Magic C18 AQ reversed-phase resin (Bioz). The column was developed with a 72 min linear gradient of acetonitrile in 0.125% formic acid delivered at 280 nL/min. MS parameter settings were as follows: MS run time = 96 min; MS1 scan range = 300.0–1500.00; Top 20 MS/MS (min. signal = 500; isolation width = 2.0; normalized coll. energy = 35.0; activation-Q = 0.250; activation time = 20.0; lock mass = 371.101237, charge state rejection enabled, charge state 1+ rejected, dynamic exclusion enabled, repeat count = 1, repeat duration = 35.0). MS/MS spectra were evaluated using SEQUEST 3 G and the SORCERER 2 v4.0 platform (Sage-N Research) (22). Searches were performed against the NCBI Rattus norvegicus database with mass accuracy of ±50 ppm for precursor ions and 1 Da for product ions. Results were filtered with mass accuracy of ±5 ppm on precursor ions and the presence of the intended modification, phospho-[s/t/y] followed by proline. The resulting data are found in Supplemental Table S1 (https://doi.org/10.6084/m9.figshare.24574411).

### Phosphoproteomic Data Analysis

**Figure 1.**
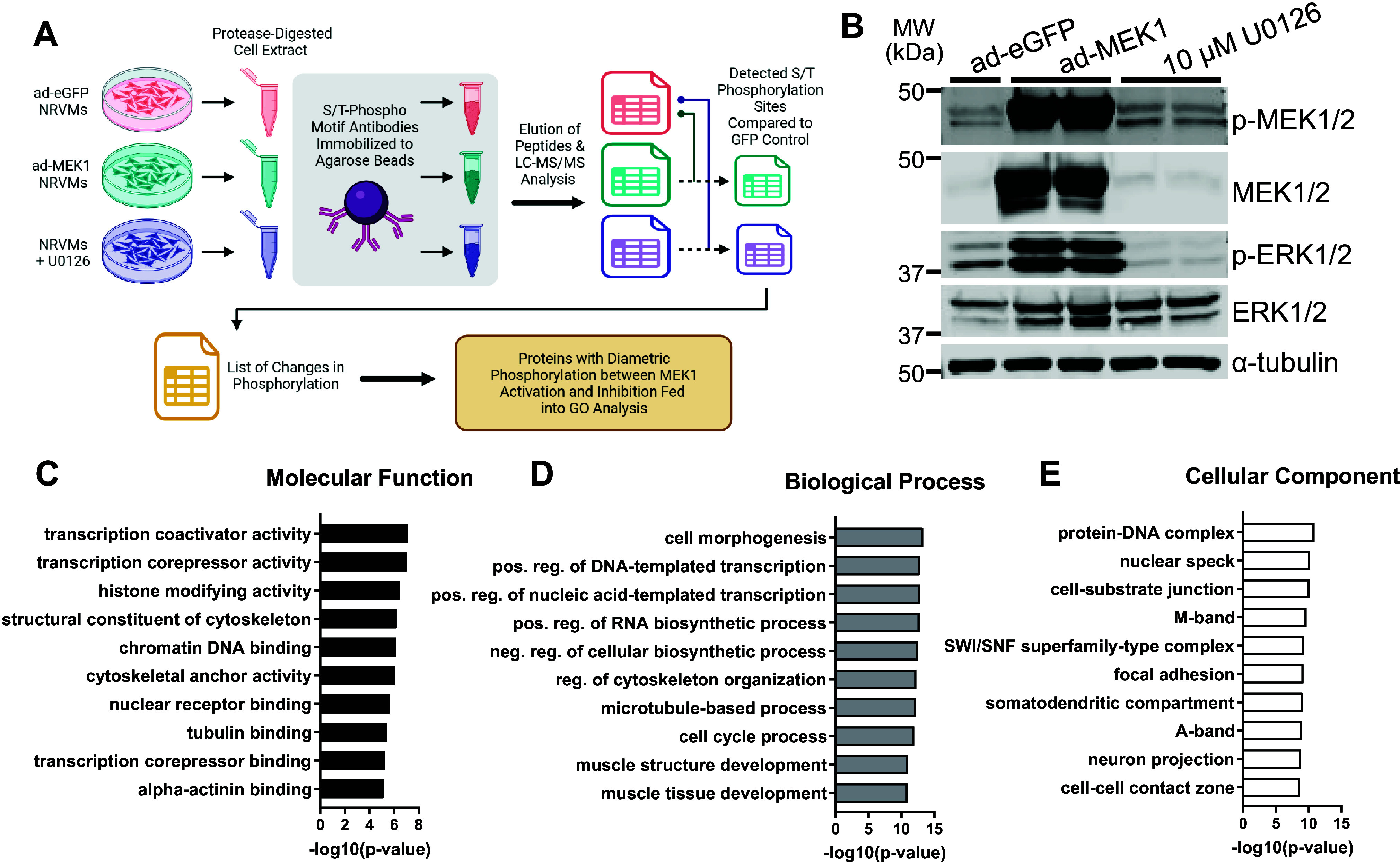
Phosphoproteomic data from analysis conducted on neonatal rat ventricular myocytes (NRVMs) with enhanced or inhibited MEK1-ERK1/2 signaling. *A*: schematic of the workflow from culture and treatment of NRVMs to the phosphoproteomic analysis (image was created with a licensed version of BioRrender.com). *B*: Western blots showing phosphorylated and total MEK1/2 and ERK1/2 with α-tubulin as a loading control for protein extracts of NRVMs subjected to enhanced green fluorescent protein (eGFP) adenovirus, activated MEK1 adenovirus, or U0126 MEK1 inhibitor, which served as inputs for the phosphoproteomics (*n* = 1–2/group with each sample representing a mixture of cells from both male and female hearts. Blots were repeated twice). *C–E*: graphs of the logarithmically transformed *P* values for gene ontology categories of the proteins identified in the screen which had phosphosites biphasically regulated under MEK1-ERK1/2 activation or inhibition for molecular function (*C*), biological process (*D*), and cellular component (*E*).

To profile upstream kinases most responsible for phosphorylation alterations associated with ad-MEK1 treatment, phosphosites were first filtered and a normalized LogFC of greater than 0.5 compared with control-treated samples was selected, which also had a negative LogFC in the inhibitor-treated samples when compared with control. Additionally, a separate list of phosphosites with a LogFC of greater than 0.5 in the inhibitor-treated samples when compared with control, with a negative LogFC in the ad-MEK1 samples when compared with control was also generated. Then, to generate a gene signature unique to the ad-MEK1 treated samples, any phosphosites that were associated with a gene present in both lists were removed from the analysis. Gene ontology was performed on genes from all phosphosites with biphasic log fold changes between the two experimental conditions using ToppFun from the Toppgene suite. The *P* values of the top 10 genes from each category were then transformed using a log10 transformation and the resulting values are represented in Fig. 1, *C–E*. Finally, the annotated upstream kinases provided in the PhosphoScan data sheet (Supplemental Table S1) for the aforementioned proteins with biphasically regulated phosphosites were tabulated and visualized in a bar graph (Supplemental Fig. S1).

### Immunofluorescence

NRVMs were cultured in 8 well chamber slides (Ibidi, Cat. No. 80826) and treated with adenoviruses or U0126, as described above. Upon completion of treatment and virus incubation, cells were washed twice in PBS, and then fixed for 15 min in 4% paraformaldehyde (Electron Microscopy Sciences, Cat. No. 15714) in PBS. Cells were then washed three times for 5 min in PBS. NRVMs were incubated in blocking solution [5% normal goat serum (Jackson Immuno, Cat. No. 005-000121), 2% bovine serum albumin (BSA), 0.2% Triton X-100] for 30 min at room temperature. Primary antibodies (Supplemental Table S2) were diluted in blocking solution and then applied to the NRVMs for 2 h at room temperature with gentle shaking on an orbital rotator. NRVMs were then washed 3× for 5 min in PBS prior to incubation with appropriate secondary antibodies (Supplemental Table S2) for 90 min at room temperature. Following three more 5-min washes in PBS, 4′,6-diamidino-2-phenylindole (DAPI; 1:2,000 in H_2_O) was applied to the samples for 10 min. DAPI was removed from the wells and cells were maintained in PBS for imaging. Secondary-only controls were conducted in concert with primary antibody experiments.

Cardiomyocytes were isolated from male and female adult mouse hearts using a postfixation collagenase digestion, as previously described (23). Briefly, hearts were excised and fixed overnight in 1% paraformaldehyde in PBS. The following day, the fixed tissue was washed in PBS for 5 min before mincing. Tissue was then incubated in 0.5 U/mL collagenase B (Roche, Cat. No. 11088815001) in 0.02% sodium azide in PBS at 37°C for 10–12 h with vigorous shaking. At the end of this period, buffer containing the dissociated cardiomyocytes was pulled off and stored at 4°C with a 2:1 ratio of 0.02% sodium azide in BGS to the digest volume. This was repeated until ventricular tissue was fully digested. To immunostain the isolated cardiomyocytes, cells were washed 3× for 5 min in PBS with centrifugations at 100 *g* for 1 min each to pellet cells and remove supernatant. Cells were incubated in blocking buffer (3% BSA, 0.2% cold water fish skin gelatin, and 0.2% Triton X-100 in PBS) for 1 h at room temperature with gentle rotation. Following centrifugation for 1 min at 100 *g*, the blocking buffer was removed, and primary antibodies (Supplemental Table S2) were diluted in modified blocking buffer [3% BSA, 0.2% cold water fish skin gelatin (Sigma, Cat. No. G7041), and 0.1% Triton X-100 in PBS] were applied. Cells were incubated in primary antibodies for 48 h at 4°C with gentle rotation. Cells were washed 3× for 5 min in PBS with centrifugations at 100 *g* for 1 min each, then secondary antibodies (Supplemental Table S2) were diluted in modified blocking buffer applied for 1 h at room temperature with gentle rotation. Following three more 5-min washes in PBS and subsequent centrifugations, cells were incubated in DAPI (1:2,000 in H_2_O) for 10 min. After one final PBS wash and centrifugation, the cardiomyocyte pellets were resuspended in 20 μL of ProLong Diamond mountant (Thermo Fisher, Cat. No. P36970) and transferred to slides with coverslips placed on top. Secondary-only controls were conducted in concert with primary antibody experiments.

NRVMs and adult cardiomyocytes were imaged using a Nikon A1 confocal microscope system, and analysis of the images was conducted with NIS Elements Advanced Research software (Nikon). For analysis of cytoplasmic actin deposition in adult cardiomyocytes, data were collected by randomly scanning samples and quantifying at least 200 cardiomyocytes.

### Histology

For histological sections of mouse hearts, tissue was fixed overnight in 10% zinc formalin, and transferred to 70% ethanol the next day before being paraffin-embedded. The paraffin blocks were sectioned at 5 µm thickness. Slides were stained with hematoxylin and eosin (H&E) and images were acquired with a Leica stereoscope (Cat. No. M165FC) equipped with a digital camera (Leica Microsystems, Cat. No. DFC310FX) and the Leica Application Suite.

### Western Blot Analysis

Cultured NRVMs were washed twice with ice-cold PBS and scraped from their dishes into ice-cold RIPA homogenization buffer, consisting of 3.5 mM SDS, 150 mM NaCl, 20 mM Tris (pH 7.4), 12 mM sodium deoxycholate, 0.5% NP-40, and 1% Triton X-100, and Halt protease and phosphatase inhibitor (Thermo Fisher, Cat. No. 78442) and vortexed for 30 s at maximum speed to lyse the cells. For ventricular lysates, hearts were excised from mice and the tissue was snap-frozen in liquid nitrogen. Ventricular tissue was then minced with scissors and homogenized using a handheld mechanical homogenizer buffer (Omni Tissue Master 125) in hypotonic lysis buffer, consisting of 10 mM NaCl, 10 mM MgCl_2_, 10 mM Tris·HCl (pH 7.5), and 0.5% NP-40, with added Halt protease and phosphatase inhibitor. Protein content for both NRVM and ventricular lysates was quantified using a bicinchoninic acid colorimetric assay (Thermo Fisher, Cat. No. 23227) and a BioTek Synergy 2 microplate reader (Agilent). Samples were made with 5× Laemmli sample buffer and denatured at 95°C for 5 min. Gels were loaded with 10 µg of NRVM lysate or 40 µg of ventricular lysate protein per lane. For traditional Western blots, after gel electrophoresis at 115 V, proteins were transferred to nitrocellulose membranes with a 0.2-μm pore size (Bio-Rad, Cat. No. 162-0112) at 85 V for 1.5 h using the standard wet transfer method. Precision Plus Protein Dual Color Standard was used as protein marker (Bio-Rad, Cat. No. 1610374), For phosphorylation analysis, 5 mM Phos-tag reagent (FUJFILM Wako, Cat. No. 304-93521) and 10 mM MnCl_2_ were added to the resolving gel mixture according to the manufacturer’s instructions. After electrophoresis at 115 V, gels were incubated in 1 mM EDTA in 1× Tris-glycine transfer buffer without methanol for 10 min with gentle rocking. Gels were then incubated in 1× Tris-glycine transfer buffer without methanol for 10 min with gentle rocking before transfer of proteins in methanol-containing transfer buffer as mentioned above. WIDE-VIEW TM-Prestained Protein Size Marker III (FUJFILM Wako, Cat. No. 230-02461) was used as a protein marker for the Phos-tag gels. Of note, migration of the bands can occur at different molecular weights in comparison to the bands of the same protein in a traditional Western blot because of the Phos-tag compound (27). Both traditional and Phos-tag membranes were blocked in 5% milk in TBST, containing 20 mM Tris (pH 7.5), 150 mM NaCl, and 0.1% (wt/vol) Tween 20 for 1 h at room temperature with shaking before being incubated with primary antibodies (Supplemental Table S2) in blocking buffer overnight on a shaker in a 4°C cold room. The next day the membranes were washed three times for 10 min each in TBST before applying appropriate Li-COR fluorescent secondary antibodies (Supplemental Table S2) for 1 h at room temperature with shaking. Following three 5-min washes in TBST, the blots were imaged on a Li-COR CLx Odyssey Imager.

### Mice and Animal Usage

*Mek1* transgenic mice [Jackson Labs (JAX) Cat. No. 010581, FVB background] expressed a cardiomyocyte-specific constitutively active form of MEK1 protein such that the MEK1-ERK1/2 signaling pathway is activated, resulting in cardiac hypertrophy (7). To genetically ablate ERK1/2 signaling in cardiomyocytes and induce cardiac dilation, germline *Mapk3^−/−^* (ERK1) mice were crossed with *loxP*-(f) targeted *Mapk1* targeted (ERK2) mice, and resultant offspring were then crossed with Myh6*-*Cre transgenic mice (JAX Cat. No. 009074, C57BL/6 background), all of which have been previously described (24–26). Wild-type *C57BL6/J* were also used (JAX Cat. No. 000664). To genotype the various mouse lines, tail samples were collected, and DNA was isolated using the Kingfisher Flex Purification system (Thermo Fisher, Cat. No. 5400610) and PCR was conducted with the primers shown in Supplemental Table S3. All mice were housed in a temperature-controlled environment with a 14-h:10-h light/dark cycle and ad libitum access to water and chow diet. All animal procedures were carried out in accordance with the Institutional Animal Care and Use Committee (IACUC)-approved protocol at CCHMC. For wild type and MEK1 transgenic cohorts, both male and female mice (2–3 mo of age and 24–36 g body wt) were used; however, for *Mapk1/3* genetically ablated and Cre-only controls, only males (2–3 mo of age and 23–31 g body wt) were used because the Myh6-Cre transgene is on the X chromosome. Randomization was not used because genetically identical mice were used between experimental and control cohorts at similar time points. Blinding of samples was performed where possible.

### Statistics

For actin lateral membrane deposition quantification, two-way ANOVAs with Tukey’s multiple comparisons tests were conducted. For intercalated disk actin deposition quantification, one-way ANOVAs with Dunnett’s multiple comparisons tests were conducted. Values of *P* < 0.05 were considered significant. Statistical analysis was conducted, and graphs were generated with Graph Pad Prism (Dotmatics). For all quantifications, values are represented as means ± SD.

## RESULTS

Cultured NRVMs were treated with an activated MEK1 adenovirus (ad-MEK1), or U0126 (a MEK1 inhibitor) and compared with NRVMs infected with an eGFP-expressing recombinant adenovirus (ad-eGFP) as a control (Fig. 1*A*). Western blotting confirmed that ad-MEK1-mediated overexpression resulted in high levels of activated MEK1 protein with increased ERK1/2 phosphorylation, while U0126 treatment reduced ERK1/2 phosphorylation (Fig. 1*B*). NRVMs were lysed and total protein extracts were collected and processed to enrich for total phosphorylated peptides for mass spectroscopy phosphoproteomic analysis, as described previously (21). Results were filtered to identify only proteins that had phosphorylation changes at the ERK1/2 consensus motif of serine/threonine-proline. The resulting data identified over 2700 combinations of amino acids on 324 proteins as changing their phosphorylation status with modulation of this signaling cascade (Supplemental Table S1). Gene ontology analysis was conducted on those proteins with altered phosphorylation sites biphasically regulated in the ad-MEK1 treated NRVMs compared with the U0126 treated. The data show a prominent upregulation of cytoskeletal categories relating to the molecular function, biological processes, and cellular components changed in the NRVMs. (Fig. 1, *C–E*). Graphical representation of the known upstream kinases of the biphasically phosphorylated proteins in the data set further confirmed that our analysis targeted known ERK1/2 phosphorylation substrates (Supplemental Fig. S1).

**Figure 2.**
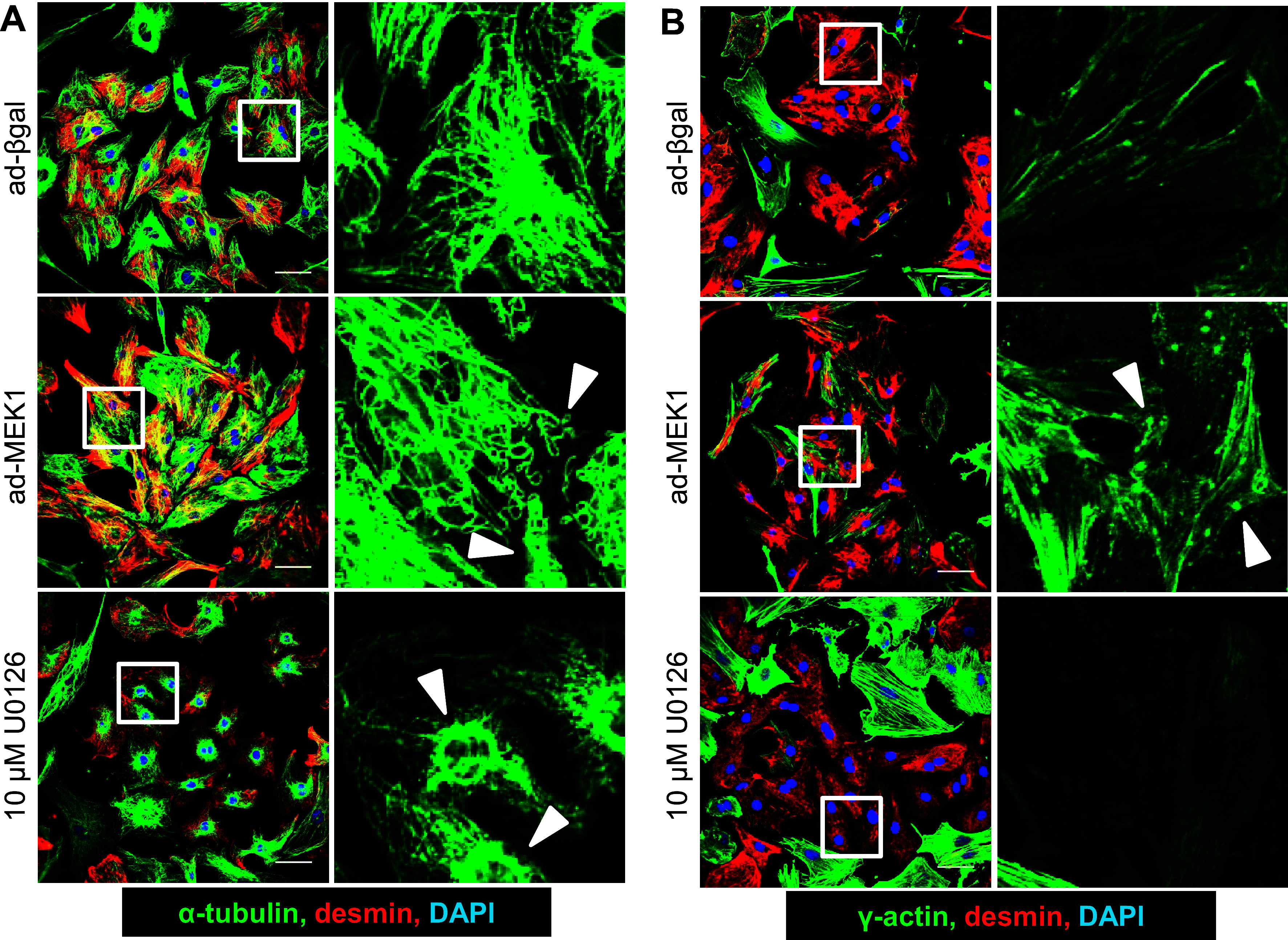
Cytoskeletal networks of neonatal rat ventricular myocytes (NRVMs) change with enhanced or inhibited MEK1-ERK1/2 signaling. *A* and *B*: representative immunocytochemistry images of cultured NRVMs treated with β-galactosidase adenovirus (ad-βGal) as control or activated MEK1 adenovirus (ad-MEK1) to activate signaling vs. 10 µM U0126 MEK1 inhibitor to block signaling. The fixed NRVM cultures were immunostained for α-tubulin (green; *A*) and γ-actin (green; *B*) and costained with desmin (red) and DAPI (blue). *Right*: each set are magnifications of their respectively associated white boxes at *left*. White arrows point to tubulin and actin structures that have changed among the conditions (NRVM cultures had a mixture of cells from both male and female hearts. Staining was repeated twice. Scale bars = 20 um).

Staining for cytoskeletal proteins in NRVMs showed that MEK1-ERK1/2 pathway modulation changed the morphology of cytoskeletal networks as analyzed by immunocytochemistry for α-tubulin, desmin, and γ-actin (Fig. 2, *A* and *B*). The desmin staining also specifically identified cardiomyocytes. With the activation of MEK1-ERK1/2 signaling, microtubules (α-tubulin) extended to the NVRM membranes in looping structures, while cytoplasmic γ-actin had a punctate staining pattern (both denoted with white arrows). With inhibition of signaling, microtubules retracted to the perinuclear space (denoted with white arrows), and expression of γ-actin was downregulated. Immunostaining of actin-interacting proteins identified in the phosphoproteomic data set, palladin and RAI14, also showed differential localization and arrangement in NRVMs with activated and inhibited MEK1-ERK1/2 signaling (Supplemental Fig. S2). With either activation or inhibition of MEK1-ERK1/2 signaling, palladin protein showed sarcomeric striations (Supplemental Fig. S2A). However, with activation of the pathway there also was movement of palladin to the nucleus, while with inhibition there was more deposition of palladin in the perinuclear space. RAI14 protein moved from the perinuclear space in control conditions to the subcortical regions of NRVMs with pathway manipulation (Supplemental Fig. S2*B*), but with pathway activation it formed punctate structures at the periphery, while with inhibition it localized in structures more akin to stress fibers.

**Figure 3.**
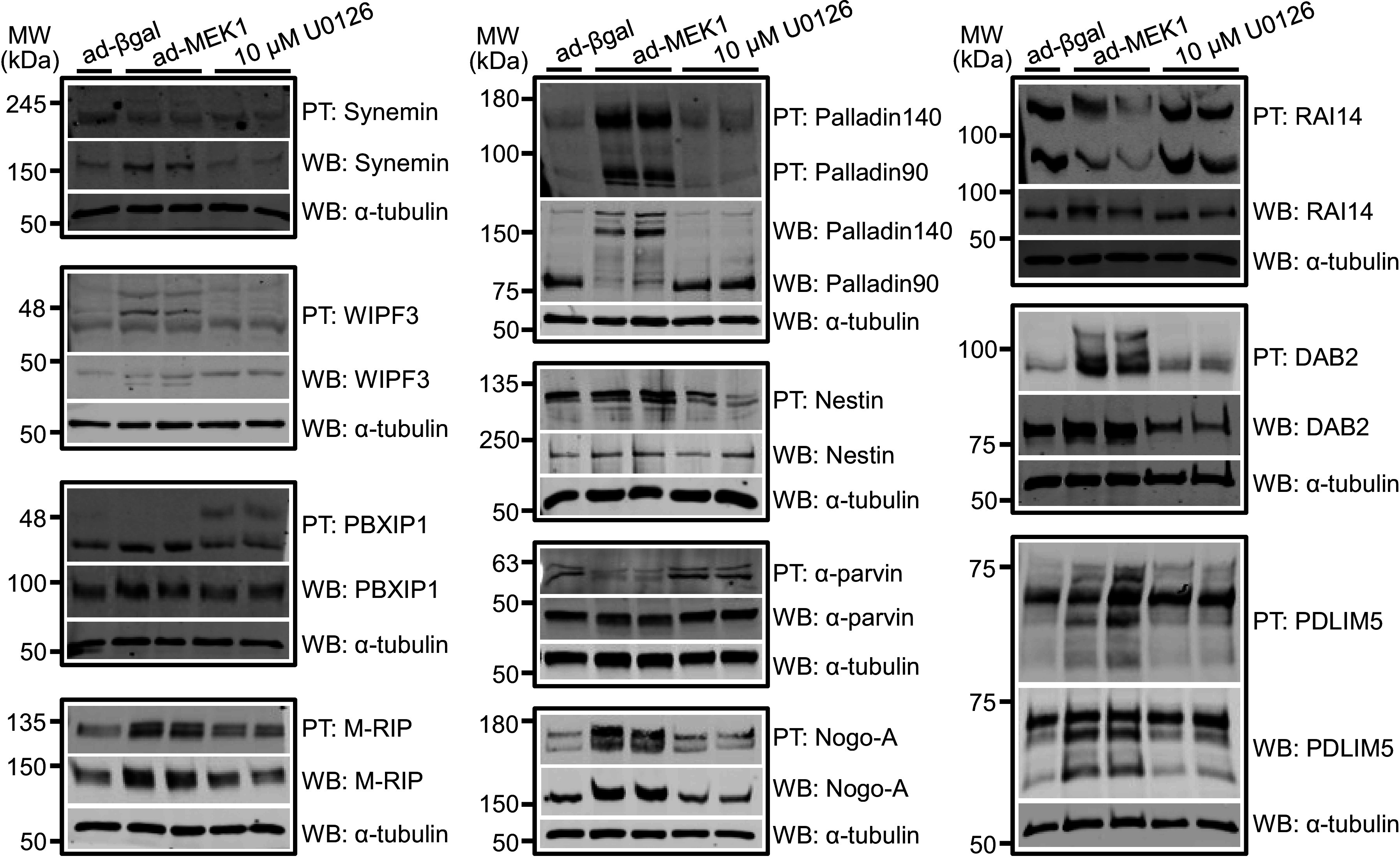
Phosphorylation and total protein levels of cytoskeletal proteins from the phosphoproteomic screen in neonatal rat ventricular myocytes (NRVMs) with activated or inhibited MEK1-ERK/2 signaling. Phos-tag (PT) and traditional Western blotting (WB) of protein lysates made from NRVMs treated prior with β-galactosidase expressing adenovirus (ad-βGal) as control or activated MEK1 adenovirus (ad-MEK1) vs. 10 µM U0126 MEK1 inhibitor were analyzed for synemin, WAS/WASL interacting protein family member 3 (WIPF3), pre-B-cell leukemia homeobox interacting protein 1 (PBXIP1), myosin phosphatase Rho-interacting protein (M-RIP), palladin, nestin, α-parvin, neurite outgrowth inhibitor (nogo-A), retinoic acid induced 14 (RAI14), disabled 2 (DAB2), and PDZ and LIM domain 5 (PDLIM5) (*n* = 1–2/group with each sample representing a mixture of cells from both male and female hearts. Blots were repeated twice). α-Tubulin Western blotting is shown as protein processing and loading control.

Phos-tag gels were used to verify and assess the phosphorylation status of the identified candidate protein targets from the screen. The Phos-tag acrylamide compound binds phosphorylated proteins and causes retardation of protein migration, although some proteins are only partially phosphorylated so multiple migratory species are not uncommon. This analysis showed that a large variety of cytoskeletal proteins and interactors were indeed changed in phosphorylation status in response to activation or inhibition of the MEK1-ERK1/2 pathway (Fig. 3). Traditional Western blotting done in tandem with Phos-tags also showed differences in total protein abundance for select proteins (Fig. 3). However, the Phos-tag compound can distort the migration of a protein in a gel resulting in the appearance of molecular weight differences between Phos-tag blots and traditional Western blots, and total protein visualization on Phos-tag blots can vary based on phosphorylation content of some bands (27). Synemin and nestin are both intermediate filament proteins (17) that displayed changes in phosphorylation. WAS/WASL interacting protein family member 3 (WIPF3), myosin phosphatase Rho-interacting protein (M-RIP), neurite outgrowth inhibitor (nogo-A), disabled 2 (DAB2), and PDZ and LIM domain 5 (PDLIM5) all have been shown to interact with or modulate actin (28–32), and similarly displayed enhanced phosphorylation with pathway activation. Other actin-binding proteins, α-parvin and retinoic acid induced 14 (RAI14) (33, 34), had reduced phosphorylation with pathway activation. Pre-B cell leukemia homeobox interacting protein 1 (PBXIP1), a microtubule binding protein (35), had enhanced phosphorylation with inhibition of MEK1-ERK1/2 signaling. Perhaps most intriguingly, the actin-binding protein palladin not only changed phosphorylation status, but also isoform expression between the 90- and 140-kDa isoforms, which has been observed previously in other cell types (36), when MEK1-ERK1/2 signaling was manipulated. Although cytoskeletal proteins were the largest differentially phosphorylated category from the data set, we also verified phosphorylation changes in both sarcomeric [leiomodin 2 (LMOD2), nebulette, myozenin 2 (MYOZ2), and synaptopodin 2-like (SYNPO2L)] and non-sarcomeric proteins (EF-hand domain 2 (EFHD2), UBX domain protein 1 [(UBXN1), LIM domain 7 (LMO7), and armadillo repeat containing X-linked 2 (ARMCX2)] identified in the phosphoproteomic data set, suggesting more broad cardiomyocyte effects (Supplemental Fig. S3).

**Figure 4.**
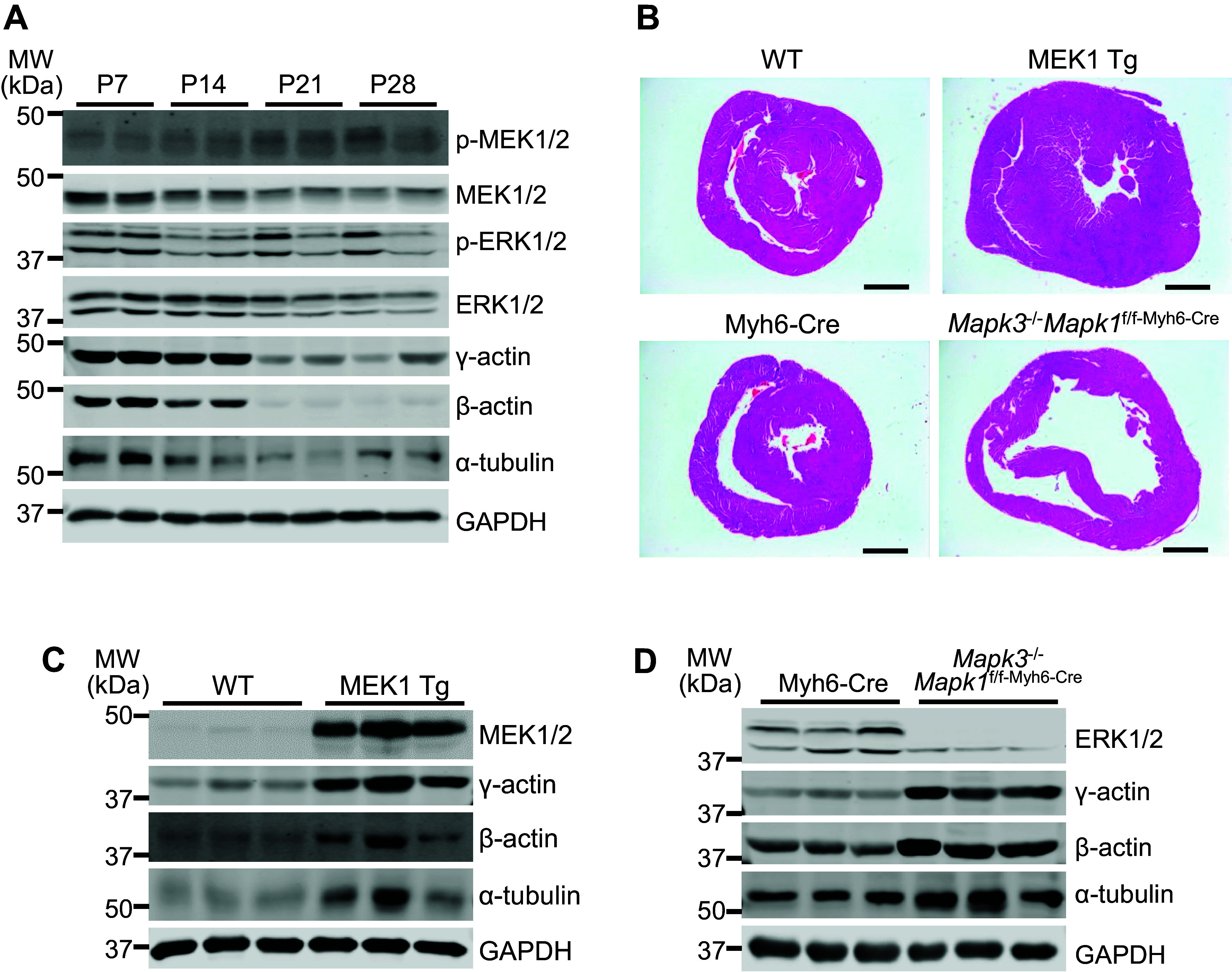
In vivo changes in cytoskeletal protein expression during development of wild type and MEK1-ERK1/2 activated and inhibited hearts. *A*: Western blots on protein lysates from whole wild type hearts of the indicated proteinsduring *postnatal days 7–28* (P7–P28; *n* = 2/time point). *B*: hematoxylin and eosin-stained histology of heart cross sections from MEK1 transgenic, *Mapk1/3* gene-deleted mice, and their respective controls. Scale bars = 1 mm. *C*: Western blots on whole heart protein lysates from 3-mo-old-activated MEK1 transgenic hearts and those of their wild type (WT) littermates for MEK1, ERK1/2, cytoplasmic actins, α-tubulin, with GAPDH as a loading control (*n* = 3/group). *D*: Western blots on whole heart protein lysates from 2-mo-old *Mapk1/3* (ERK1/2) genetically ablated hearts (*Mapk3*^−/−^*Mapk1*^f/f-Myh6-Cre^) and those of *Myh6*-Cre-only controls for MEK1, ERK1/2, cytoplasmic actins, α-tubulin, with GAPDH as a loading control (*n* = 3/group and blots were repeated twice).

**Figure 5.**
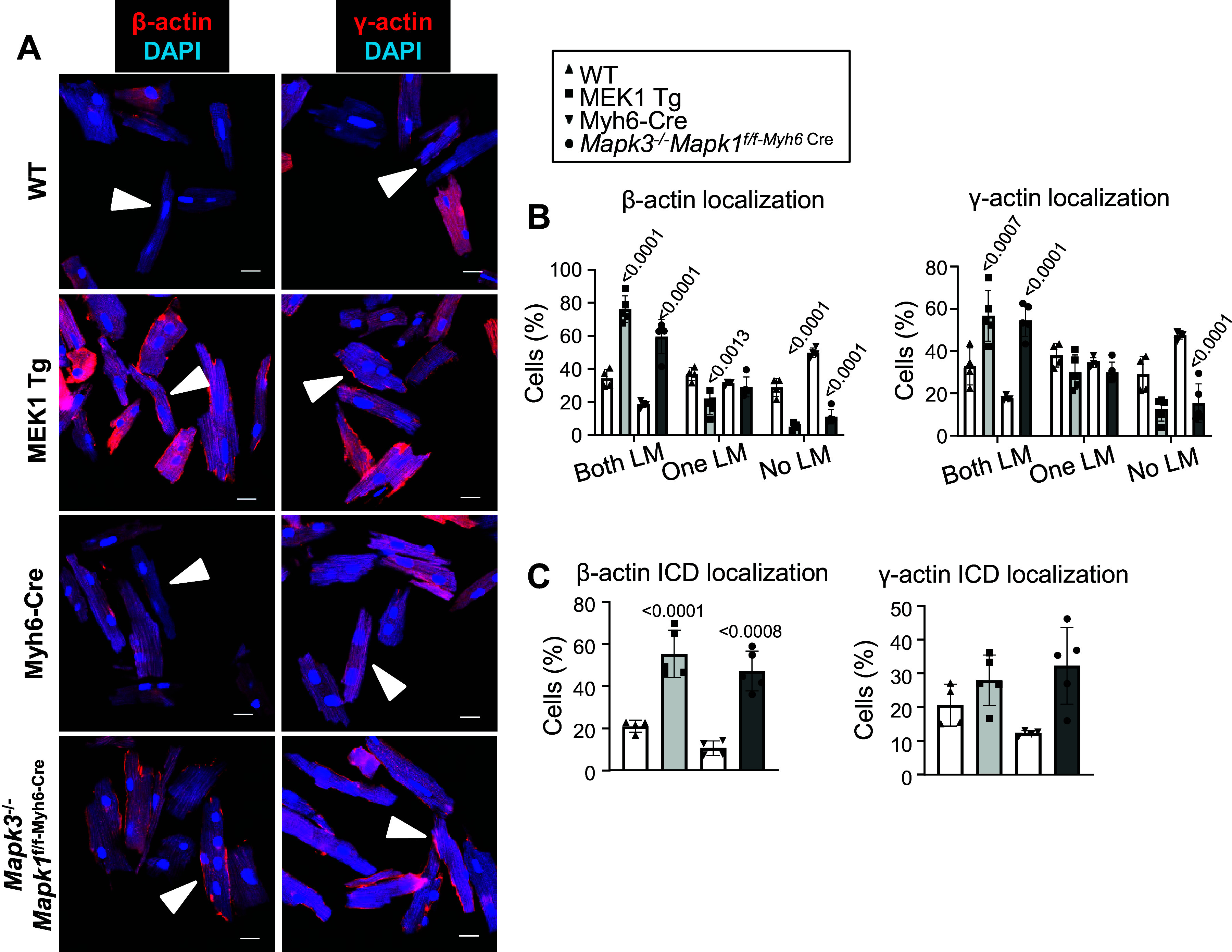
Localization of β-actin and γ-actin in adult cardiomyocytes isolated from male and female activated MEK1 transgenic, male *Mapk1/3* gene-deleted mice, and their respective controls. *A*: representative images of immunocytochemistry staining of cytoplasmic actin localization in red with DAPI nuclear staining in blue (white arrows highlighting the subcortical distribution of both isoforms; scale bars = 10 µm). *B* and *C*: graphs showing the quantification of both isoforms at the lateral membranes (LMs; *B*) and intercalated discs (ICDs; *C*) of isolated myocytes (*n* = 4–5/group). Statistical significance was determined by 1-way ANOVA with appropriate post hoc tests. Error bars represent standard deviation and exact *P* values for the genetically manipulated mice as compared with their respective controls are listed above the bars.

With evidence that MEK1-ERK1/2 signaling is associated with altered phosphorylation of the non-sarcomeric cytoskeleton of NRVMs in vitro, we also examined how this pathway was modulated in the hearts of mice. Interestingly, MEK1/2, ERK1/2, β-actin, γ-actin, and α-tubulin were most highly expressed in the *postnatal day 7* (P7) wild-type heart, but each of these were downregulated after P14 through P28 (Fig. 4*A*). Using adult (2–3 mo old) hearts from MEK1 transgenic and *Mapk1/3* (ERK1/2) genetically ablated mice, which develop hypertrophic and dilated hearts, respectively (Fig. 4*B*), we observed that the cytoplasmic actin proteins and α-tubulin are upregulated (Fig. 4, *C* and *D*). More intriguingly, this induction is also mirrored in immunostaining of cytoplasmic actin in adult cardiomyocytes from these mice. In contrast to previous studies (14, 15), we observed that the β-actin and γ-actin localized to the subcortical space under the sarcolemma of cardiomyocytes of all genotypes (white arrows, Fig. 5*A*). However, upon quantifying the percentage of cells with actin deposition at both, one or none of the lateral membranes (LM), we found that MEK1 transgenic and *Mapk1/3* genetically deleted cardiomyocytes have enhanced deposition of both actin isoforms in the lateral membranes (Fig. 5*B*). In addition, the data revealed that cardiomyocytes from both MEK1 transgenic and *Mapk1/3* genetically deleted mice showed increased β-actin, but not γ-actin, at their intercalated disks compared with controls (Fig. 5*C*), suggesting the potential for differential roles for these two actins between the lateral membranes and intercalated disks. It was interesting that both increased or decreased MEK1-ERK1/2 signaling in vivo had the same effect on induction of β-actin and γ-actin in the heart and their subcortical localization patterns (Fig. 5, *B* and *C*). Of note, we did observe some minor localization of the β-actin and γ-actin in a striated pattern when cardiomyocytes grew in width (MEK1 transgenic) or length (ERK1/2 genetically ablated) when imaging in two dimensions (Fig. 5*A*), as have previous reports (14, 15). However, upon rendering the adult cardiomyocytes in three dimensions, we found that very little was associated with the sarcomeres, hence the vast majority of the cytoplasmic actins remain in the subcortical space and not as part of the sarcomeres.

## DISCUSSION

Here we have found that components of the cardiomyocyte cytoskeleton, particularly the non-sarcomeric actin network, are modified by changes in MEK1-ERK1/2 signaling, a link that has been described in other cell types, but less understood in cardiomyocytes. Cardiomyocytes appear to be the only cell type described thus far as having a directional growth switch linked to the upregulation or downregulation of MEK1-ERK1/2 signaling (6). This may mean that these kinases have binding partners or downstream effectors unique to cardiomyocytes that allow them to mediate the directionality of growth. Indeed, the MEK1-ERK1/2 pathway is a known effector of mitotic growth in many proliferative cell types (37); however, as adult mouse cardiomyocytes lack proliferative capacity, it is likely that this pathway has been specialized to modulate growth dynamics.

It has been previously proposed that the phosphorylation of costamere proteins is a main determinant of directional growth in cardiomyocytes (38). Indeed, muscle cells sense tension through both muscle myosin and nonmuscle myosin-based contractions (39), and tension is a driver of both concentric and eccentric cardiac growth (8). Here, we show that a variety of cytoskeletal proteins are potential effectors of MEK1-ERK1/2 signaling, suggesting the hypothesis that cytoskeletal modifications contribute to how new sarcomeres are deposited in either series or parallel within the cardiomyocyte. However, there is little information in the current literature that delineates the function of the cytoplasmic actin network, especially how it might affect growth dynamics during development or with disease-dependent remodeling. One previous correlative study showed upregulation of β-actin in feline cardiomyocytes in response to right ventricular pressure overload (14), but further studies on cardiomyocyte-specific cytoplasmic actin dynamics are lacking. Microtubules have been suggested to regulate structure and signaling in cardiomyocytes (16). Much less data are available on the function of cytoplasmic actin isoforms in these cells, despite the fact that cross talk between microtubules and actin networks has been shown, and multiple actin nucleating, capping, and depolymerization proteins are known to underlie genetic cardiomyopathies (18, 40).

The past literature is also unclear as to the intracellular localization of β-actin in adult cardiomyocytes. One examination of β-actin in feline cardiomyocytes used a GFP-tagged version of the protein and found high sarcomeric deposition (14). However, GFP is known to spuriously bind to sarcomeric myosin (41). When the authors stained for endogenous β-actin, they found much less of it within the sarcomeres, and instead, lateral cardiomyocyte membrane localization was observed (14). Another study using immunogold labeling of cytoplasmic actin in transmission electron microscopy showed β-actin at the z-disks, while γ-actin projected from the top and bottom ends of z-disks and was postulated to interact with mitochondria (15).

In skeletal muscle, γ-actin appears to reside within the subcortical space under the sarcolemma where it interacts with costamere proteins (13). Our analysis here shows that cytoplasmic β/γ-actins localize primarily to the subcortical space in a cardiomyocyte, similar to γ-actin in skeletal muscle myocytes. This subcortical localization of cytoplasmic actin likely plays an important role in sarcolemmal membrane signaling, given that in skeletal muscle deletion of the gene encoding β-actin produced a mild myopathy in the quadriceps with weakened function (42). Interestingly, we observed more β-actin than γ-actin at the intercalated disks of cardiomyocytes (Fig. 5), which may suggest some divergence in function between these two isoforms in cardiomyocytes. In other cells, γ-actin tends to be more ubiquitously expressed, while β-actin has more definitive localization to cell protrusions (11, 43). Furthermore, a recent cryoEM study found differences among the NH_2_-terminal structures of the four sarcomeric and cytoplasmic actins, suggesting specificity between these actin isoforms in both function and their interactions with accessory proteins (44).

Intriguingly, we observed an upregulation of both β-actin and γ-actin during increased and decreased activity of the MEK1-ERK1/2 pathway in adult hearts of activated MEK1 transgenic and *Mapk1/3* gene-deleted mice. It is interesting to speculate that the appearance of the cytoplasmic actin network within the subsarcolemmal space could participate in growth signaling by facilitating placement and regulation of the protein production machinery to allow new sarcomeres in either series or parallel, which might also underlie dilated and concentric remodeling in select forms of human heart disease. Indeed, we observed upregulated expression of the cytoplasmic actin network in hypertrophic and dilated mouse hearts, consistent with the hypothesis that they might play a role in the addition of new sarcomeres or in cellular remodeling. Thus, we propose that the reemergence of a prominent cytoskeletal actin network in the subcortical compartment, especially along the lateral cardiomyocyte membrane, may play an important structural and signaling roles in the transduction of MEK1-ERK1/2-based growth within cardiomyocytes.

## DATA AVAILABILITY

Data will be made available upon reasonable request.

## SUPPLEMENTAL DATA

10.6084/m9.figshare.24574411 Supplemental Tables S1–S3 and Figs. S1–S3: https://doi.org/10.6084/m9.figshare.24574411.

## GRANTS

This work was supported by National Heart, Lung, and Blood Institute Grants 1F32HL138747 (to K.M.G.) and 5R01HL105924 (to J.D.M.).

## DISCLOSURES

No conflicts of interest, financial or otherwise, are declared by the authors.

## AUTHOR CONTRIBUTIONS

K.M.G. and J.D.M. conceived and designed research; K.M.G., M.M., and S.L.K.B. performed experiments; K.M.G., M.M., S.L.K.B., and J.D.M. analyzed data; K.M.G. and J.D.M. interpreted results of experiments; K.M.G. and J.D.M. prepared figures; K.M.G. and J.D.M. drafted manuscript; K.M.G., M.M., C.O.S., S.L.K.B., D.P.M., and J.D.M. edited and revised manuscript; K.M.G., M.M., C.O.S., S.L.K.B., D.P.M., and J.D.M. approved final version of manuscript.
